# Letter to the editor—a need to strengthen the adverse drug reaction reporting during COVID-19

**DOI:** 10.1186/s43162-021-00070-z

**Published:** 2021-11-09

**Authors:** Satvinder Singh Bakshi, Sumita Bakshi

**Affiliations:** 1 Department of ENT and Head & Neck Surgery, AIIMS Mangalagiri, Post box 522503, Guntur, Andhra Pradesh India; 2 Dr Smilez Dental Clinic, Pondicherry, 605001 India

To the editor,

Since the outbreak of the coronavirus 2019 (COVID-19) pandemic, the loss of life has been immense. Physicians have struggled to treat patients and have found them helpless in saving the lives of those affected. Such desperate times have created an environment where many known drugs have been tried for treating the infection. Many times, this has resulted in more harm than benefit; for example, one of the first therapies to be explored was chloroquine and hydroxychloroquine. This drug has long been used for malaria and inflammatory diseases. Many case studies and trials showed promise early on; however, there was minimal data on dosing regimen for effectiveness and safety. Eventually, in a randomized control trial, hydroxychloroquine treatment was shown to have no decrease in mortality and actually showed a higher incidence of mechanical ventilation and death [1].

Adverse drug reactions (ADRs) can be defined as “an appreciably harmful or unpleasant reaction resulting from an intervention related to the use of a medicinal product; adverse effects usually predict hazard from future administration and warrant prevention, or specific treatment, or alteration of the dosage regimen, or withdrawal of the product” [2]. They are one of the leading causes of hospital admissions and prolonged morbidity in patients in developed countries [2]. Many ADRs are underreported mainly because physicians and other support staff are unaware or do not consider it important to report [3].

Other drugs like antivirals/antiretrovirals like lopinavir have also been tried. Many times, the dosing of these drugs is above the standard dosing for the main disease they are meant for; this implies that the chance of adverse drug reactions is also high. Other drugs currently being used are ivermectin, doxycycline, azithromycin, and remdesivir [4]; in addition, multivitamins like vitamin C and vitamin D_3_ have also been advocated. The data on the adverse reaction of these drugs in COVID patients is limited; besides, since multiple medicines are being used, it is likely that the drug–drug interactions will also be higher. In their study, Crescioli G et al. reported unexpected and uncommon psychiatric ADRs in hospitalized patients with COVID-19 infection; they also stressed on the burden of drug–drug interactions and the need to report them [5]. Some of the drug interactions reported by them include azithromycin with lopinavir, atorvastatin and warfarin and lopinavir with metformin, and levothyroxine [5].

At present, there is a paucity of any new drugs for tackling this infection; hence, we have to depend on these drugs for the time being. It is therefore imperative that physicians and other medical staff be sensitized to the need of reporting any ADRs in patients with COVID-19 infection. This will help build a good database and direct future research in the development of an efficacious and safe drug regimen for the management of COVID-19 infection. We also suggested that the patients themselves should be educated and sensitized to look for and report any adverse event they experience following the use of these drugs.

“Do no harm” or non-maleficence is the cornerstone of our medical practice, and adequate reporting of ADRs will go a long way in achieving the same.

## Data Availability

Not applicable

## Abbreviations

COVID-19: Coronavirus 2019
ADR: Adverse drug reaction
